# Health-Aware Food Recommendation Based on Knowledge Graph and Multi-Task Learning

**DOI:** 10.3390/foods12102079

**Published:** 2023-05-22

**Authors:** Yi Chen, Yandi Guo, Qiuxu Fan, Qinghui Zhang, Yu Dong

**Affiliations:** 1 Beijing Key Laboratory of Big Data Technology for Food Safety, Beijing Technology and Business University, Beijing 100048, China; ddyyg@st.btbu.edu.cn (Y.G.); fanqx_19@st.btbu.edu.cn (Q.F.); zhangqinghui@st.btbu.edu.cn (Q.Z.); 2 School of Computer Science, University of Technology Sydney, Sydney, NSW 2008, Australia

**Keywords:** health, food recommendation, knowledge graph, graph convolution network, multi-task learning

## Abstract

Current food recommender systems tend to prioritize either the user’s dietary preferences or the healthiness of the food, without considering the importance of personalized health requirements. To address this issue, we propose a novel approach to healthy food recommendations that takes into account the user’s personalized health requirements, in addition to their dietary preferences. Our work comprises three perspectives. Firstly, we propose a collaborative recipe knowledge graph (CRKG) with millions of triplets, containing user–recipe interactions, recipe–ingredient associations, and other food-related information. Secondly, we define a score-based method for evaluating the healthiness match between recipes and user preferences. Based on these two prior perspectives, we develop a novel health-aware food recommendation model (FKGM) using knowledge graph embedding and multi-task learning. FKGM employs a knowledge-aware attention graph convolutional neural network to capture the semantic associations between users and recipes on the collaborative knowledge graph and learns the user’s requirements in both preference and health by fusing the losses of these two learning tasks. We conducted experiments to demonstrate that FKGM outperformed four competing baseline models in integrating users’ dietary preferences and personalized health requirements in food recommendations and performed best on the health task.

## 1. Introduction

A nutritious diet matters as the cornerstone of good health, and unhealthy dietary habits can lead to a host of preventable illnesses. According to a statistical report [1] from the World Health Organization in 2016, over 1.9 billion adults and 41 million children under the age of five were clinically overweight, with 650 million living with obesity. Poor dietary habits [2], particularly diets high in calories, sugar, and fat, and low in fiber, are primarily responsible for this epidemic. Improving the dietary structure is considered an effective approach to tackling this issue [3]. However, making healthy dietary choices can be challenging for individuals due to their hectic lifestyles and limited knowledge of food and nutrition.

In recent years, with the blossoming research in the food field [4,5,6,7], food recommendation systems [8] have garnered considerable attention from the public, promising tailor-made food choices to users [9,10,11]. However, current systems are constrained by two main factors. Firstly, they frequently overlook crucial food-related information such as recipe ingredients, food categories, and dietary restrictions when building recommendation models. This omission can significantly impede the system’s ability to establish connections between users and foods, resulting in less accurate and varied recommendations. Secondly, most algorithms disregard users’ health needs when defining their objectives, relying solely on dietary preferences. For instance, conventional food recommendation systems will offer recommendations based on a user’s preference for high-fat and high-sugar diets, irrespective of potential health risks. Thus, food recommendation systems must account for both dietary preferences and health requirements to offer effective recommendations [12,13].

In this paper, we propose a novel health-aware food recommendation model, namely FKGM, which leverages a knowledge graph and multi-task learning approach. The FKGM takes into account both the user’s dietary preferences and the health impact of four critical nutrients in recipes: sodium, fat, sugar, and saturated fat. By integrating these considerations into the recommendation process, FKGM offers users personalized food recommendations that are in line with their dietary preferences and health requirements.

The main contributions of this study are summarized into three oriented aspects as follows:

- Data-oriented: We created a collaborative recipe knowledge graph (CRKG) by merging the user–recipe bipartite graph and the recipe knowledge graph [14]. The CRKG includes user–recipe interactions and food-related details such as ingredients, food types, and cuisines. The CRKG provides the foundation of the recommendation model with attribute-based and association-based information on users and recipes, enabling the model to acquire a more comprehensive understanding of users and recipes that better reflects their semantic similarity.
- Score-oriented: We defined a score-based method for evaluating the healthiness match between recipes and user preferences. First, we defined a Nutrient Content Score in recipes and a user Nutrient Intake Score based on the nutrition criteria recommended by the UK Food Standards Agency to assess the healthiness of recipes and user preferences, respectively. We then defined a Nutrient Discrepancy Score between recipes and users to determine the healthiness match between recipes and user preferences, which is used to calculate the loss of the health learning task in the multi-task learning layer of the recommendation model.
- Model-oriented: We developed a new health-aware food recommendation model, FKGM, which embeds entities and relationships on the collaborative recipe knowledge graph using TransD [15] and updates entity representations through message passing using a knowledge-aware attention graph convolutional neural network. The model was trained using multi-task learning, which involves preference learning and health learning tasks. Our experimental results demonstrate that FKGM outperformed four existing baseline models in striking a balance between preference needs and health requirements.

The remainder of this paper is organized as follows. Section 2 describes related work, Section 3 introduces the construction of the collaborative knowledge graph, Section 4 introduces the methods for evaluating recipe and user preferences, Section 5 presents the proposed model, Section 6 details the experimental setup and results, and Section 7 provides a summary of this study.

## 2. Related Work

In this section, we first review knowledge graph-based recommendation methods, then review preference-based and health-aware food recommendation methods. In the field of food recommendation, preference-based and health-aware methods have their own advantages and limitations. Therefore, it is a worthwhile and challenging problem to effectively combine the two and balance users’ preferences and health. Consequently, the work of our paper focuses on food recommendation that combines user preferences and health requirements.

### 2.1. Knowledge Graph-Based Recommendation

Knowledge graphs organize entities and relations in the real world in the form of graphs, which can effectively express the semantic relations between entities and have been applied to recommendation systems. The methods of knowledge graph-based recommendation systems mainly fall into two categories: path-based methods and embedding-based methods.

**Path-based methods.** These methods mainly use paths in knowledge graphs to make recommendations, which can intuitively represent users’ preference propagation. Hu et al. [16] proposed a context-aware recommendation method based on meta-paths, which uses knowledge graphs to describe the relationships between users and items and uses meta-paths to capture these relationships. Fan et al. [17] proposed a meta-path-guided heterogeneous graph neural network for intent recommendation tasks. However, these methods usually rely heavily on the design of meta-paths.

**Embedding-based methods.** These methods usually use knowledge graph embedding [18] to learn a low-dimensional representation vector for each entity and relation in knowledge graphs, while preserving the structural information of knowledge graphs [19,20,21], and then use the vectors obtained by knowledge graph embedding to enhance the representation of users or items in recommendation systems. Zhang et al. [22] proposed CKE, which uses TransR for knowledge graph embedding and extracts semantic features from structured knowledge to enrich item representation. Many studies in embedding-based methods use graph neural networks to leverage their powerful modeling ability for graph data to improve the recommendation performance. Wang et al. [23] proposed KGAT, which explicitly models high-order connections in knowledge graphs in an end-to-end manner and attempts to use message-passing mechanisms to exploit structural knowledge for the first time. Ma et al. [24] addressed the problems of error propagation and weak interpretability in recommendation systems that combine graph neural networks and knowledge graphs and proposed KR-GCN, which designed a transition-based triplet scoring method and introduced a path-level self-attention mechanism to distinguish the contributions of different selection paths and predict interaction probabilities, achieving results better than baselines.

Our model belongs to embedding-based methods, which use knowledge graph embedding to learn entity and relation embeddings and use the message passing mechanism of a graph neural network to capture high-order relational information for entities and update entity representation.

### 2.2. Preference-Based Food Recommendation

In this section, we review the existing methods for preference-based food recommendation, which aim to recommend foods to users based on their taste and dietary preferences. We categorize these methods into three main types: collaborative filtering, content-based, and hybrid methods. However, each method has its strengths and weaknesses, and there are also challenges and open problems for future research.

**Collaborative filtering methods.** These methods learn user preferences from user–food interactions, such as ratings and tags, and assume that users who have similar interactions with foods will have similar preferences. For example, Ge et al. [25] combined user ratings and tags in food recommendations and used matrix factorization to generate food recommendations. Khan et al. [26] proposed a feature recognition technique based on EnsTM to effectively model user preferences. Collaborative filtering methods can capture the diversity and personalization of user preferences, but they also suffer from data sparsity and cold start problems, which limit their scalability and applicability. Therefore, it is important to explore other methods to overcome these limitations.

**Content-based methods.** These methods use food content to learn user preferences, such as ingredients and food images, and assume that users will prefer foods that have similar content to the foods they have liked before. For example, Freyne et al. [27] decomposed recipes into individual ingredients and constructed user profiles composed of ingredients that the user likes based on recipe ratings containing these ingredients, thus improving the recommendation performance. Yang et al. [28] incorporated food image information into food recommendations and learned user preferences based on food image content. Their work demonstrated the importance of food image information in learning food preferences. Content-based methods can overcome the data sparsity and cold start problems of collaborative filtering methods, but they also face the challenges of extracting and representing food content features, as well as dealing with the semantic gap between low-level features and high-level preferences. In addition, these methods may not capture the diversity and novelty of food recommendations as effectively as collaborative filtering methods.

Hybrid methods. These methods combine both collaborative filtering and content-based methods to leverage their advantages and mitigate their disadvantages. Gao et al. [29] considered food recommendation as a multimedia task and proposed a hierarchical attention-based method, HAFR, which simultaneously considers factors such as user–recipe interactions, food images, and food ingredients in modeling. Experimental results show that the average recommendation performance of the proposed method is 12% better than that of the baseline model. Gao et al. [30] considered the correlations between a user–recipe, recipe–ingredient, and ingredient–ingredient, providing richer auxiliary information for food recommendation models and helping to better model user preferences. They used graph convolutional neural networks to model the relationships between these foods. This method outperformed the state-of-the-art method in the recommendation performance by 5.4%. Consequently, hybrid methods have become a popular approach for food recommendation due to their ability to capture diverse and personalized user preferences.

Compared to the above methods, our method further considers the complex relationships between foods and uses knowledge embedding and a knowledge graph attention network to model the vector representations of entities in the food knowledge graph. In this way, the model can fully utilize the rich semantic information contained in CRKG, explore users’ high-level preferences, and increase recommendation diversity. However, there are also challenges in designing and optimizing knowledge-based models, such as dealing with the sparsity and noise in knowledge graphs, as well as the complexity of the model training and inference process. Therefore, future research can focus on addressing these challenges and developing more effective and efficient knowledge-based food recommendation methods.

### 2.3. Health-Aware Food Recommendation

The healthy food recommendation based on the user’s health status, nutritional needs, and dietary restrictions aims to recommend beneficial foods or recipes to improve users’ health levels and quality of life. However, it may not consider users’ personal preferences and diversity. There are two main methods for existing healthy food recommendations: rule-based and data-driven.

**Rule-based methods.** These methods rely on specific rules [31] or criteria, such as filtering, sorting, or classification, often limited by the usage scenario and based on domain knowledge, expert opinions [32], or logical reasoning. Shandilya et al. [33] proposed a food recommendation system named MATURE-Food that recommends suitable foods based on users’ current mandatory requirements, using a real food item dataset [34] and medical records of chronic kidney disease (CKD) patients from University of Iowa Hospitals and Clinics (UIHC), mainly for medical and health fields rather than general food recommendation scenarios. Ribeiro et al. [35] presented a mobile catering recommendation system named SousChef, targeting elderly people and creating personalized meal plans based on users’ information including body measurements, personal preferences, and activity levels. The nutritional suggestions and application are designed for elderly people, featuring a user-friendly interface and following the guidelines of nutritionists. User testing was conducted to determine the applicability of recipes and nutrition plans and the usability of the mobile application. The results showed that more than 70% of elderly participants were satisfied with the simplicity of the meal plan recommendations and the SousChef application. Ge et al. [36] considered calorie balance and proposed a calorie balance function based on the difference between user’s needs and recipe calories, where users can define whether food recommendations should focus more on taste or health. However, rule-based methods typically use general or fixed rules without considering users’ personalized needs.

**Data-driven methods.** With the development of the data era, data-driven methods are becoming mainstream. These methods can utilize a large amount of data to learn users’ preferences and needs and generate more accurate and personalized recommendations based on them. They usually use machine learning or statistical techniques to analyze data and extract useful information from it. Wang et al. [37] proposed a personalized health food recommendation scheme. The scheme consists of three parts: the recipe retrieval, user health analysis, and health food recommendation. The authors describe users’ health conditions by capturing text health-related information crawled from social networks, and propose a novel recommender based on category-aware hierarchical memory networks to learn health-aware user–recipe interactions, to better perform food recommendations. Chen et al. [38] proposed a new framework called NutRec, which aims to provide users with healthy recipe recommendations by simulating the interactions and proportions of ingredients in recipes. The framework consists of three main parts: ingredient prediction, ingredient quantity prediction, and healthy recipe recommendation. The authors conducted experiments on two recipe datasets, and the results supported the intuition of the framework and demonstrated its ability to retrieve healthier recipes. Li et al. [39] introduced a novel approach to recipe recommendation that incrementally shifts users towards healthier recipe options while respecting their past preferences. The authors proposed a model that jointly learns recipe representations via a graph over two graphs extracted from a large-scale Food KG, capturing different semantic relationships across the preferences and healthiness aspects. Experimental results on two large real-world recipe datasets showcase the model’s ability to recommend tasty as well as healthy recipes to users. However, this approach only considers the healthiness of the food itself and does not take into account users’ personalized health requirements.

Our work belongs to the data-driven method. Compared with the methods mentioned above, our method can represent the complex relationship between users and food more comprehensively by learning rich semantic information on CRKG. Furthermore, our method simultaneously learns user preferences and personalized health requirements. By providing corresponding constraints in the recommendation process, our work aims to offer food recommendations that not only satisfy users’ tastes but also meet their personalized health requirements.

## 3. Collaborative Recipe Knowledge Graph

In this paper, the collaborative recipe knowledge graph (CRKG) refers to a collaborative knowledge graph that combines the user–recipe bipartite graph and recipe knowledge graph. It contains user–recipe interaction relationships and various food-related information, such as recipe–ingredient and recipe–cuisine relations. This provides food recommendation models with a better understanding of the semantic relationships between users and recipes and allows for the exploration of implicit associations between users and recipes. To clarify the construction process of CRKG, we first provide a formal description of the user–recipe bipartite graph, recipe knowledge graph, and CRKG, and then introduce the method for constructing CRKG.

### 3.1. Formal Definition

- **User–Recipe Bipartite Graph** $G_{1}$**:** We defined the set of m users as $U=\{u_{1},u_{2},\ldots ,u_{m}\}$ and the set of n recipes as $RP=\{rp_{1},rp_{2},\ldots ,rp_{n}\}$. The user–recipe interaction data are represented as a bipartite graph $G_{1}=\{\left(u,interact,rp\right)|u\in U,rp\in RP\}$, where $interact_{u,rp}=1$ if there exists an interaction history between user u and recipe rp, and $interact_{u,rp}=0$ otherwise.
- **Recipe Knowledge Graph** $G_{2}$**:** The constructed recipe knowledge graph is represented as $G_{2}=\left\{\left(h,r,t\right)|h\in E,r\in R,t\in E\right\}$, which consists of many entity-relation-entity triplets $\left(h,r,t\right)$, where h,r,t, respectively, represent the head entity, relation, and tail entity, describing the relationship between the head entity h and the tail entity t through the relation r. For instance, consider the triplet is $\left(pizza,food.dish.ingredients,mozzarella\;cheese\right)$. In this case, ‘pizza’ is the head entity, ‘food.dish.ingredients’ is the relation, and ‘mozzarella cheese‘ is the tail entity. This triplet indicates that mozzarella cheese is one of the ingredients of pizza. E and R represent the sets of entities and relations in the recipe knowledge graph $G_{2}$, respectively.
- **Collaborative Recipe Knowledge Graph** CRKG**:** We integrated the user–recipe bipartite graph $G_{1}$ and the recipe knowledge graph $G_{2}$ into a unified graph CRKG. Specifically, for any recipe rp∈RP in $G_{1}$, a corresponding entity e∈E can be found in $G_{2}$. For example, Roast Chicken with Rosemary is a recipe in both $G_{1}$ and $G_{2}$. $G_{1}$ and $G_{2}$ are aligned and fused into a unified graph CRKG based on the matched recipes, denoted as $CRKG=\left\{\left(h,\;r,\;t\right)|h\in E\prime ,r\in R\prime ,t\in E\prime \right\}$, where $E^{\prime }=E\cup U$ and $R=R^{\prime }\cup \{interact\}$.

### 3.2. Construction Method

In this study, we first constructed a user–recipe bipartite graph $G_{1}$ using the widely used public dataset Allrecipes for food recommendation. The Allrecipes dataset contains user–recipe interaction data, ingredients, and nutritional information. We extracted user–recipe interaction data from Allrecipes to construct the user–recipe bipartite graph $G_{1}$, which includes 48,111 users, 38,115 recipes, and 1,267,176 interactions between them.

Then, the recipe knowledge graph $G_{2}$ was constructed using the large-scale knowledge base Freebase to provide recommendation models with recipe-related relational information. Freebase [40] is a large-scale knowledge base consisting of nodes and edges, where each node represents an entity (such as a person, place, organization, etc.), and each edge represents a relationship between two entities. It was proposed by Google in 2007 and covers knowledge in various fields such as arts, history, and food. It has been widely used in research on knowledge graphs, natural language processing, intelligent question answering, and other fields. This study used food-related knowledge from Freebase to construct the recipe knowledge graph $G_{2}$. Specifically, entity linking technology was used to construct $G_{2}$. This process first linked food entities in the Allrecipes dataset to their corresponding entities in Freebase and extracted the other entities that the corresponding entity is linked to in Freebase and the relationships between them to obtain triplets to form $G_{2}$. Then, the obtained triplets were refined by filtering out low-frequency relations and entities. Finally, a recipe knowledge graph $G_{2}$ was constructed, which contains 69,799 entities, 10 types of relations, and 2,500,801 triplets. $G_{2}$ includes 10 food-related relationships, encompassing various aspects such as the food dish-type (recipe-type), compatibility of ingredients with dietary restrictions (recipe–dietary restrictions), cuisine of food dishes (recipe–cuisine), ingredient compatibility with dietary restrictions (ingredient–dietary restrictions), narrower categorization of ingredients (ingredient categories), cuisine associated with an ingredient (ingredient–cuisine), dishes served with a specific dish (served-with), regions associated with a dish (region), and recipe–ingredient relationship.

**Figure 1:**
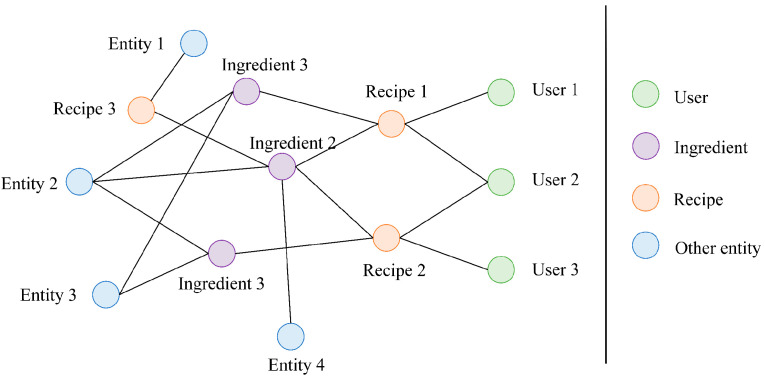
A toy example of collaborative recipe knowledge graph. The bipartite graph formed by connecting the user nodes and recipe nodes in Figure 1 is denoted as $G_{1}$, while the graph formed by the recipe nodes and the remaining nodes is denoted as $G_{2}$.

After obtaining the user–recipe bipartite graph $G_{1}$ and recipe knowledge graph $G_{2}$, the two are fused into CRKG, as shown in Figure 1; the nodes of different colors in the figure represent different types of entities in CRKG. The green node represents a user, the orange node is a recipe, and the connection between the user and the recipe indicates that there is an interaction between them. In the figure, the recipe–ingredient association relation is shown as an example. The purple nodes are recipe ingredient nodes, and the recipe nodes and ingredient nodes are connected to indicate that the recipe is composed of these ingredients. The blue nodes are other types of entities in the collaborative knowledge graph. The nodes in CRKG are called entities, and the edges between entities are represented as relations.

## 4. Assessment of Recipe and User Preference Healthiness

In this section, we first propose a calculation method for assessing the healthiness of recipes and the healthiness of user preferences and then define a method for calculating the healthiness match between recipes and user preferences.

### 4.1. Recipe Nutrient Content Score (NCS)

Before making healthy food recommendations, a standard needs to be developed to measure the healthiness of a recipe. In this paper, we refer to the nutrient rating system proposed by the UK Food Standards Agency (FSA) [41] to evaluate the healthiness of recipes. This rating assesses the healthiness of a recipe based on the content of four nutrients: sodium, fat, sugar, and saturated fat, as the long-term excessive intake of any of these nutrients can lead to health risks. We defined the set of these four nutrients as the nutrient set=sodium,fat,sugar,saturatedfat. The FSA rating evaluates the nutrient content of recipes and classifies them into three levels: healthy, medium, and unhealthy. Table 1 shows the healthy threshold values for the four nutrients (the salt content is converted to sodium content using a conversion factor of 0.388 g of sodium per 1 g of salt), and a recipe is considered unhealthy if the content of any nutrient exceeds its corresponding threshold value.

In this study, the nutrient content of each recipe was obtained from Allrecipes and Table 2 shows examples of the nutrient content of several recipes in the dataset.

In this study, the Nutrient Content Score $\left(NCS\right)$ of a recipe was defined with reference to the FSA rating. The healthiness of a recipe was assessed based on the content of the recipe on four nutrients, which was calculated as shown in Equation (1). $NCS_{k}\left(rp\right)$ denotes the Nutrient Content Score of recipe rp on the k-th nutrient. k is the k-th nutrient in the nutrient set k∈nutrient set. $threshold_{k}$ is the limit value of nutrient k out of the health range in the FSA rating. $content\left(rp,k\right)$ is the content value of nutrient k in recipe rp.

(1) $${NCS}_{k}\left(rp\right)=\frac{content\left(rp,k\right)\times 10}{{threshold}_{k}}$$

The NCSs of a recipe calculated by Equation (1) for the four nutrients are continuous values and unified to the same scale. Table 3 shows the NCSs calculated by Equation (1) for the recipes in Table 2. According to the NCS scoring system, the scores can be categorized into three intervals: the healthy range is defined as [0,4), the moderate range is defined as [4,10), and the unhealthy range is defined as [10,+∞).

### 4.2. User-Preferred Nutrient Intake Score (NIS)

The core of this study’s recommendation task is to recommend healthier recipes based on users’ dietary habits. Since different users have different dietary habits, their health requirements are also different. Users’ dietary habits reflect not only their dietary preferences but also their health status. Therefore, we defined the Nutrient Intake Score to calculate users’ high and low intake levels in four nutrient categories. In calculating the Nutrient Intake Score (NIS), a grouped weighted average method is used instead of directly averaging a certain nutrient in the user’s interaction history to effectively avoid the influence of extreme values. The formula for calculating the Nutrient Intake Score for a certain nutrient is shown in Equation (2), where k∈nutrient set.

(2) $${NIS}_{k}\left(u\right)=\frac{c_{1}\times {med}_{1}+c_{2}\times {med}_{2}+c_{3}\times {med}_{3}}{Count}$$

The specific calculation process is as follows:

**Input**: User’s recipe history interaction data.

**Procedure**:

Step 1. First, all the recipes in the user’s historical recipe interaction data are counted, and they are divided into three groups based on the NCS of each recipe in nutrient k, where [0,4) is the low level group, [4,10) is the medium level group and [10,+∞) is the high level group.

Step 2. Calculate the frequency of recipes in each group and calculate the median NCS in each group. The frequency of each group is denoted as $c_{1}$, $c_{2}$, and $c_{3}$; the median NCS of recipes in each group is denoted as ${med}_{1}$, ${med}_{2}$, and ${med}_{3}$; and Count is the total number of user recipe interaction records.

Step 3. Apply Equation (2) to obtain ${NIS}_{k}\left(u\right)$.

**Output**: User’s Nutrient Intake Score in nutrient k.

The NIS is calculated for the user in terms of sodium, fat, sugar, and saturated fat according to Equation (2). Typically, if a user’s score for a certain nutrient intake is high, it indicates that the user has consumed too much of that nutrient in their diet, and therefore restrictions should be added to that nutrient in food recommendations.

### 4.3. Nutrient Discrepancy Score (NDS)

After defining the calculation method for recipe healthiness and user preference for healthiness, we defined the Nutrient Discrepancy Score (NDS) of a recipe for a user, which measures the degree of match between a recipe and a user in terms of healthiness. It is calculated based on the Nutrient Intake Score of the user and the Nutrient Content Score of the recipe. The higher the NDS, the less suitable the recipe is for the user’s health.

The specific calculation process is as follows:

**Input**: Nutrient Content Score of recipe rp, Nutrient Intake Score of user u.

**Procedure**:

Step 1. Modifying the original softplus function [42] as shown in Equation (3), the modified function will map values less than four to close to zero. Using this equation, the values of each item in NCS and NDS are mapped, and small values are mapped to values close to zero, while larger values are preserved. The purpose of this modification is to focus on the unhealthy nutrients in NCS or NIS, and ignore the healthy nutrients, when calculating the Nutrient Discrepancy Score between the recipe and the user in the next step. The Nutrient Content Score ${NCS}_{k}\left(rp\right)$ of recipe rp and the Nutrient Intake Score ${NIS}_{k}\left(u\right)$ of user u are processed using Equation (3).

(3) $$softplus_{m}od\left(x\right)=log\left(1+e^{x-4}\right)$$

Step 2. Based on the $softplus_{m}od\left({NIS}_{k}\left(u\right)\right)$ and $softplus\_mod({NCS}_{k}(rp))$ obtained above, the Nutrient Discrepancy Score $NDS\left(u,rp\right)$ of recipe rp and user u is calculated as shown in Equation (4). When the NIS of a user on a certain nutrient is equal to 10 and the NCS of a recipe on a certain nutrient is equal to 10, the NDS of both is calculated by Equation (4) ≈ 36. Therefore, in general, when the $NDS\left(u,rp\right)$ exceeds 36, the recipe is considered unfavorable to the health of the user.

(4) $$NDS\left(u,rp\right)=\sum_{k\in nutrient\;set}softplus_{m}od\left({NIS}_{k}\left(u\right)\right)\times softplus_{m}od\left({NCS}_{k}\left(rp\right)\right)$$

**Output**: Nutrient Discrepancy Score (NDS) of recipe rp for user u, denoted as $NDS\left(u,rp\right)$.

The following is an example to illustrate the calculation method of *NDS*. Assume that the Nutrient Intake Scores of two users ($u_{1}$ and $u_{2})$ and the Nutrient Content Scores of two recipes (${rp}_{1}$ and ${rp}_{2}$) are known, as shown in Table 4.

Table 5 shows the results of users $u_{1}$ and $u_{2}$ with recipes ${rp}_{1}$ and ${rp}_{2}$, respectively, to calculate the Nutrient Discrepancy Scores.

From Table 5, it can be analyzed that for user $u_{1}$, recipe ${rp}_{1}$ is not conducive to their health, while recipe ${rp}_{2}$ is more suitable for user $u_{1}$. This is because user $u_{1}$ has an excessive intake in terms of fat and has unhealthy eating habits, while recipe ${rp}_{1}$ is too high in terms of fat content and is shown to be unhealthy, while recipe ${rp}_{2}$ is low in fat content. For user $u_{2}$, both recipes ${rp}_{1}$ and ${rp}_{2}$ are not conducive to their health. This is because user $u_{2}$ has unhealthy dietary habits in terms of sodium and sugar, and recipe ${rp}_{1}$ is too high in sodium and is shown to be unhealthy, while recipe ${rp}_{2}$ is too high in sugar and is also shown to be unhealthy. Therefore, both recipe ${rp}_{1}$ and recipe ${rp}_{2}$ have a high *NDS* for user $u_{2}$.

## 5. Recommendation Model FKGM

We define the task of personalized healthy food recommendation as follows: given the CRKG, the goal is to learn a prediction function $F\left(u,rp|\Theta ,CRKG\right)$ with parameters Θ that can capture both the user’s preference and health requirements, and output the matching degree between user u and recipe rp.

### 5.1. Preliminary

Before delving into the details of our proposed model, we introduce the data preprocessing steps and how the model works in this section. This will help readers understand the underlying processes.

When constructing the positive and negative sample sets for user preference, for each user, the recipes that have been interacted with are considered as the positive sample set $P_{u}^{+}$, while the recipes that have not been interacted with are considered as the negative sample set $P_{u}^{-}$.

When constructing the positive and negative sample sets for user health, the NDS calculation method proposed in Section 4 is first applied to calculate the NDS between all users and recipes in the recommendation dataset. Based on the NDS values between the user and the recipe, the positive and negative sample sets for healthy recipes, $H_{u}^{+}$ and $H_{u}^{-}$, are defined for each user. If the NDS between a user and a recipe is greater than or equal to 36, the recipe is added to the negative sample set $H_{u}^{-}$ for that user. The positive and negative sample sets for user health defined here will be used for the health learning task of the model.

The multi-task learning stage of FKGM learns the user’s preference and health needs through the preference learning task and the health learning task. In both learning tasks, the Bayesian Personalized Ranking [43] loss is used as the loss function [44] to optimize the model parameters. The optimization goal is to make the model’s recommendation results closer to the recipes in the positive sample set. In deep learning, the loss function is a function that measures the gap between the model’s prediction results and the true results. During the training process, the optimization algorithm adjusts the model’s parameters continuously, making the value of the loss function gradually decrease, thereby making the model’s prediction results closer and closer to the target results.

**Figure 2:**
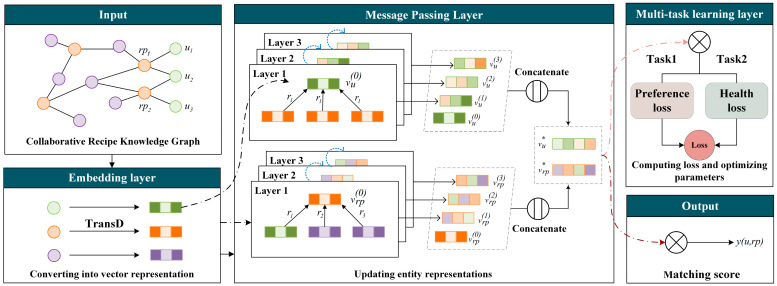
Illustration of the proposed FKGM model. The two curves outputted by the message passing layer in the graph, with the light pink curve representing the calculation of inner products between entities for computing loss during the model training phase, and the dark red curve representing the calculation of inner products between entities for outputting predicted matching scores during the model inference phase.

The overall framework and operation process of the model is introduced below. The framework of the healthy food recommendation model FKGM is illustrated in Figure 2, which consists of three components: (1) the embedding layer, which vectorizes each entity and relation in CRKG into a vector through knowledge graph embedding; (2) the message passing layer, which utilizes a knowledge-aware attention graph convolutional neural network to perform iterative updates on the vector representation of each entity by receiving messages from its neighborhood; and (3) the multi-task learning layer, performing the preference learning task and health learning task, respectively.

The input of the FKGM is CRKG constructed in Section 3. Firstly, in the embedding layer, all the entities and relations on the CRKG are embedded into vector [45] forms so that the computer can better understand and process this data. The vectors corresponding to entities or relations are also known as their representation. Then, in the message passing layer, each entity on the CRKG iteratively receives information from its neighboring entities that are connected to itself and assigns different weights to the information from different neighboring entities according to the relation attention coefficients. This process is also known as message passing. Through multiple layers of message passing, each entity can obtain extensive neighborhood information, thereby better capturing the complex relations in the graph structure. Figure 2 shows an example of three-layer message passing, where an entity is updated to a new representation after each layer of message passing. The final representation of an entity is obtained by concatenating its vector representation before message passing and its vector representations after each layer of updating.

Then, the model predicts the matching score between a user and a recipe by taking the inner product of vectors [46], a method commonly used in recommendation or classification tasks to calculate the similarity between two vectors. During the optimization phase of the model, which is the multi-task learning layer, the preference learning task calculates the loss of the model in terms of preference prediction and the health learning task calculates the loss of the model in predicting health requirements. The two losses are then weighted and summed to obtain the final loss used for optimizing the model parameters.

### 5.2. Embedding Layer

**Figure 3:**
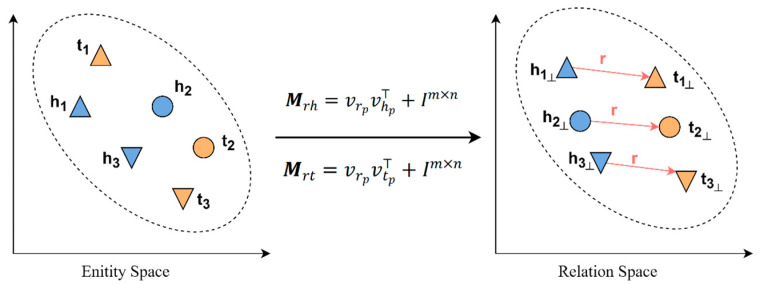
Simple illustration of TransD.

Embedding is the process of transforming entities and edges into vectors, which are usually called embedding vectors. Embedding vectors can map entities and edges from their original symbolic form (such as strings or IDs) to a continuous vector space, which facilitates computation and processing. We first used xavier_uniform to initialize the vectors of entities and relations. Then we used knowledge graph embedding to update the vector representations. Knowledge graph embedding aims to learn the latent representations of entities and relationships in the knowledge graph while preserving the structural information of the graph. CRKG is a heterogeneous graph with rich semantic information. We adopted TransD [15] as the method of knowledge graph embedding, which embeds entities and edges in CRKG into continuous vector space while retaining its structural information. TransD employs a method of dynamically constructing mapping matrices. Given a triplet $\left(h,r,t\right)$, its vector includes $v_{h}$, $v_{h_{p}}$, $v_{r}$, $v_{r_{p}}$, $v_{t}$, and $v_{t_{p}}$, where the subscript p indicates the projected vector, $v_{h}$, $v_{h_{p}}$, $v_{t}$, $v_{t_{p}}\in R^{d}$ and $v_{r}$, $v_{r_{p}}\in R^{z}$. As shown in Figure 3, TransD maps the head entity h and tail entity t into a common space constructed by the entity and relation in the triplet using two mapping matrices, $M_{rh}$ and $M_{rt}$.

The head entity h and tail entity t are mapped to the relation space by constructing the projection matrix, as shown in Equation (5).

(5) $$v_{h_{⊥}}=M_{rh}v_{h},\;v_{t_{⊥}}=M_{rt}v_{t}$$

The symbols $v_{h_{⊥}}$ and $v_{t_{⊥}}$ in Equation (5) indicate the representations of the head entity h and the tail entity t in the relation space, respectively. The plausibility score $g\left(h,r,t\right)$ of a triplet is defined by Equation (6). TransD uses embedding scores to measure the plausibility of a triplet’s embedding in a knowledge graph. The embedding score of a triplet $\left(h,r,t\right)$ is defined by Equation (6), where a higher score indicates a more plausible embedding and a lower score indicates a less plausible one. The TransD scoring function first projects the head and tail entities into vector spaces that correspond to the relation and then calculate the distance between the projected head entity and the projected tail entity plus the relation vector. The smaller the distance, the higher the score, indicating the plausibility of the triplet. In other words, if the projected head entity is close to the projected tail entity plus the relation vector, then it is more likely that the triplet $\left(h,r,t\right)$ is valid in the knowledge graph.

(6) $$g\left(h,r,t\right)=-{\left\|v_{h⊥}+v_{r}-v_{t⊥}\right\|}_{2}^{2}$$

TransD adopts the Bayesian Personalized Ranking loss, which aims to maximize the margin between positive and negative samples. The loss function is shown in Equation (7).

(7) $$L_{kg}=\sum_{\left(h,r,t\right)\in \varepsilon^{+}}\sum_{\left(h,r,t^{\prime }\right)\in \varepsilon^{-}}-ln\sigma \left(g\left(h,r,t^{\prime }\right)-g\left(h,r,t\right)\right)$$

In Equation (7), $\varepsilon^{+}$ represents the set of true triplets in the CRKG, which has positive samples, while $\varepsilon^{-}$ represents the negative samples. Negative samples are constructed by randomly selecting an entity $t^{\prime }$ to replace the true tail entity t in the triplet $\left(h,r,t\right)$. σ denotes the sigmoid function.

### 5.3. Message Passing Layer

In this layer, the model constructs a knowledge-aware attention graph convolutional neural network, which updates entity representations through message passing [47]. In the message passing layer, each entity in CRKG broadcasts its representation to its first-order neighborhood entities that are directly connected to it and receive information from its neighborhood entities. The first-order neighborhood of an entity refers to the entities that are directly connected to it, while the high-order neighborhood refers to the entities that are indirectly connected to it. In one layer of message passing, all entities in CRKG integrate the received neighborhood information to generate their new representations. By stacking multiple layers of such operations, each entity can receive information from high-order neighborhoods, thus capturing the high-order similarity between entities. Inspired by KGAT [23], we introduced a knowledge-aware attention mechanism in the message-passing process, which can better distinguish the influence of different relations on entities in CRKG.

The first stage of message passing is to receive information from neighboring entities. The computation method for propagating neighborhood information for an entity e on CRKG is defined as Equation (8).

(8) $$v_{N\left(e\right)}=\sum_{\left(r,t\right)\in N\left(e\right)}\pi_{r}\left(e,t\right)v_{t}$$

Here, $v_{N\left(e\right)}$ represents the neighborhood information of entity e, $N\left(e\right)$ represents the set of all neighboring entities of entity e, $\;v_{t}$ denotes the representation of the neighboring entity t, and $\pi_{r}\left(e,t\right)$ is the normalized relation attention coefficient between entity e and entity t under relation r. When entity e and entity t are mapped to relation space r and their distance is closer to r, it indicates that entity t is more important to entity e under relation space r. In this step, the representations of all neighboring entities of entity e are multiplied by the normalized relation attention coefficients and summed to obtain the neighborhood information of entity e. The definition of relation attention coefficient ${\hat{\pi }}_{r}\left(e,t\right)$ is shown in Equation (9).

(9) $${\hat{\pi }}_{r}(e,t)=(v_{t_{⊥}}{)}^{T}tanh(v_{e_{⊥}}+v_{r})$$

We used the softmax function to normalize the relation attention coefficients for all neighboring entities connected to entity e, as shown in Equation (10). The relation attention coefficient distinguishes the different importance levels of neighboring entities when entity *e* receives their information.

(10) $$\pi_{r}\left(e,t\right)=\frac{\exp\left({\hat{\pi }}_{r}\left(e,t\right)\right)}{\sum_{\left(r^{\prime },t^{\prime }\right)\in N\left(e\right)}\exp\left({\hat{\pi }}_{r^{\prime }}\left(e,t^{\prime }\right)\right)}$$

After obtaining all the information from the neighborhood of entity e, the next step is to aggregate the information. In this step, the neighborhood information of entity e and the representation of entity e itself are aggregated to update the representation of entity e. We adopted the bi-interaction approach to aggregate the neighborhood information, which can comprehensively capture and process the interaction information between entities. The specific aggregation formula is shown in Equation (11).

(11) $$f_{Aggregator}=LeakyReLU\left(W_{1}\left(v_{e}+v_{N\left(e\right)}\right)\right)+LeakyReLU\left(W_{2}\left(v_{e}⊙v_{N\left(e\right)}\right)\right)$$

The matrices $W_{1},W_{2}\in R^{d}$ are trainable parameter matrices, and the ⊙ operator denotes element-wise multiplication between vectors. The LeakyReLU activation function was used. After one layer of message passing, the entity representation is updated, which is abstracted as Equation (12).

(12) $$v_{e}^{\left(l\right)}=f\left(v_{e}^{\left(l-1\right)},v_{N\left(e\right)}^{\left(l-1\right)}\right)$$

Here, $v_{e}^{\left(l\right)}$ represents the representation of entity e in the l-th layer of message passing. After one layer of message passing, the entity updates its representation based on the aggregation function and neighborhood information, enabling it to obtain information from the first-order neighborhood. By stacking more layers, entities on graph CRKG can capture information from higher-order neighborhoods, and thus mine users’ latent preferences.

After L layers of message passing, entities on CRKG are updated to a new representation at each layer. The final representation of entities on CRKG is obtained by concatenating their representations across all layers.

(13) $$v_{e}^{*}=v_{e}^{\left(0\right)}||\ldots ||v_{e}^{\left(L\right)}$$

The model outputs the predicted matching score by calculating the inner product between the user entity representation $v_{u}^{*}$ and the recipe entity representation $v_{rp}^{*}$. The larger the inner product between the user entity representation $v_{u}^{*}$ and the recipe entity representation $v_{rp}^{*}$, the higher the degree of match between them predicted by the model.

(14) $$y\left(u,rp\right)=v_{u}^{*T}\cdot v_{rp}^{*}$$

### 5.4. Multi-Task Learning Layer

The task of the FKGM model is to learn the user’s preferences and health requirements, so two learning tasks are set at the multi-task learning layer, namely the preference learning task and the health learning task.

#### 5.4.1. Health Learning Task

In Section 4.3, we define the Nutrient Discrepancy Score of a recipe for a user, where a higher $NDS\left(u,rp\right)$ means that in terms of health, recipe rp is less suitable for user u. Based on the Nutrient Discrepancy Score, we defined a healthy positive sample set $H^{+}$ and a healthy negative sample set $H^{-}$ for each user. In the context of the health learning task, it is anticipated that the value of $y\left(u,rp\right)$ will be higher when the recipe rp is better aligned with the health requirements of user u, and conversely, lower when the recipe is less suitable for user u’s health needs. We used the idea of the Bayesian Personalized Ranking loss, and for the health learning task, the loss function $L_{health}$ is shown in Equation (15).

(15) $$L_{health}=\sum_{\left(u,{rp}_{h}^{+}\right)\in H^{+},\left(u,{rp}_{h}^{-}\right)\in H^{-}}-ln\sigma \left(\left(NDS\left(u,{rp}_{h}^{-}\right)-NDS\left(u,{rp}_{h}^{+}\right)\right)\left(y\left(u,{rp}_{h}^{+}\right)-y\left(u,{rp}_{h}^{-}\right)\right)\right)$$

In Equation (15), $NDS\left(u,rp_{h}^{+}\right)$ and $NDS\left(u,rp_{h}^{-}\right)$ represent the NDS between user u and recipe $rp_{h}^{+}$ and between user u and recipe $rp_{h}^{-}$, respectively. σ is the sigmoid function. Recipe $rp_{h}^{+}$ is randomly selected from user $u^{’}s$ healthy positive sample set $H^{+}$, while recipe $rp_{h}^{-}$ is randomly selected from user $u^{’}s$ healthy negative sample set $H^{-}$. For user u, the NDS of recipes in $H^{+}$ is lower than that of recipes in $H^{-}$. The difference in NDS between the healthy positive sample $rp_{h}^{+}$ and the healthy negative sample $rp_{h}^{-}$ is used as the weight for the score difference of the predicted matching between the healthy positive and negative samples. When the difference in NDS between the two samples is larger, the match between these two recipes and user $u^{’}s$ health requirements is further apart, and the loss in the health learning task is greater.

#### 5.4.2. Preference Learning Task

For the preference learning task, the expectation is that $y\left(u,rp_{p}^{+}\right)$ should be greater than $y\left(u,rp_{p}^{-}\right)$ when there exists an interaction between user u and recipe $rp_{p}^{+}$ but not between user u and recipe $rp_{p}^{-}$. We used the Bayesian Personalized Ranking loss as the loss function $L_{preference}$ to optimize the model parameters, which is formulated as Equation (16).

(16) $$L_{preference}=\sum_{\left(u,{rp}_{p}^{+}\right)\in P^{+},\left(u,{rp}_{p}^{-}\right)\in P^{-}}-ln\sigma \left(y\left(u,{rp}_{p}^{+}\right)-y\left(u,{rp}_{p}^{-}\right)\right)$$

In Equation (16), $P^{+}$ is the set of positive samples of user interactions, i.e., the interactions between user u and recipe ${rp}_{p}^{+}$ that exist in the interaction history. $P^{-}$ is the set of negative samples of user interactions, i.e., the interactions between user u and recipe ${rp}_{p}^{-}$ that do not exist.

#### 5.4.3. Multi-Task Loss Combination

The final objective function is a weighted combination of $L_{health}$ and $L_{preference}$, as defined in Equation (17), where $\lambda_{h}$ is the health loss weight of the health learning task and $\lambda {\left\|\Theta \right\|}_{2}^{2}$ is the regularization term [48] used to prevent overfitting.

(17) $$Loss=\lambda_{h}L_{health}+L_{preference}+\lambda {\left\|\Theta \right\|}_{2}^{2}$$

We used mini-batch Adam [49] to optimize the prediction loss and update the model’s parameters. Adam is an algorithm used for gradient descent optimization, typically used for training deep learning models. Adam has the characteristic of an adaptive learning rate and performs well in handling large-scale datasets and high-dimensional parameter spaces.

## 6. Performance Evaluation

We evaluated the proposed healthy food recommendation model, aiming to answer the following questions.

- RQ1: How does the performance of the FKGM compare to several baseline models in balancing the performance on both preference and health requirements in the food recommendation task?
- RQ2: How should the health loss weight of the health learning task be set to balance the preference needs and health requirements?
- RQ3: How do different hyper-parameters affect the performance of the FKGM model?

### 6.1. Datasets and Baselines

We conducted experiments on the recipe dataset Allrecipes, which has been described in Section 3. The experimental dataset includes 48,111 users, 38,115 recipes, and 1,267,176 interactions between them. The interaction history of each user was divided into a training set and a test set according to a 9:1 ratio.

We compared our proposed FKGM with four baseline models on the task of healthy food recommendation.

**CKE [22]**: Collaborative knowledge base embedding is a recommendation algorithm that combines collaborative filtering with knowledge graph embedding. It uses TransR to extract information from the knowledge graph and extract semantic features from structured knowledge.

**CFKG** [50]: Collaborative filtering with knowledge graphs is a recommendation model that enhances the accuracy of recommendations by integrating collaborative filtering and knowledge graphs. It maps the relationships among users, relations, and items to a triplet prediction to achieve more precise recommendations.

**KGAT [23]**: A knowledge graph attention network is a recommendation model tailored to knowledge-aware personalized recommendation. Built upon the graph neural network framework, KGAT explicitly models the high-order relations in collaborative knowledge graphs to provide better recommendations with item side information.

**BPRMF** [43]: Bayesian Personalized Ranking matrix factorization is a recommendation algorithm based on matrix factorization, which uses the Bayesian Personalized Ranking loss function to consider both the similarity of users’ interests and the relative level of interest in items.

The hyper-parameters setting of FKGM is presented in Table 6.

### 6.2. Evaluation Metrics

To evaluate the effectiveness of the model’s recommendation results in meeting users’ preferences and health requirements, separate evaluation metrics were set for preference and health requirements.

For preference evaluation, we used the commonly used recommender system evaluation metric, *Recall*. The equation is shown as Equation (18).

(18) $$Recall=\frac{\sum_{u\in U}\left|R\left(u\right)\cap T\left(u\right)\right|}{\sum_{u\in U}T\left(u\right)}$$

$R\left(u\right)$ represents the set of recipes recommended to a user, and $T\left(u\right)$ represents the set of recipes that the user has interacted with in the test set. *Recall* [46] measures the proportion of recommended recipes in the final recommendation list that the user has interacted with in the past, reflecting the relevance of the recommended results to the user’s historical interaction list. The higher the recall value, the higher the relevance of the recommended results. *Recall*@K represents the recall value of the top K recommended recipes.

The goal of this study for evaluating users’ health requirements is to constrain the recommendations provided by the food recommendation model for users who have excessive intakes of nutrients such as sodium, fat, sugar, and saturated fat to achieve nutritional balance. To evaluate whether the model can meet users’ health requirements, we ranked users based on their Nutrient Intake Scores in sodium, fat, sugar, and saturated fat in the dataset. In each ranking group, we selected the top 500 users, which were then divided into four groups: high-sodium, high-fat, high-sugar, and high-saturated fat. These four groups represent populations with excessive nutrient intake in the corresponding areas and should reduce their intake of these nutrients. This paper will use the average content of the corresponding nutrients in the top K recommended recipes generated by the model as the evaluation metric, in grams, represented as Sodium@K, Fat@K, Sugar@K, and Saturated Fat@K, respectively, to evaluate the recipes recommended to these four groups of users. The lower the value of this metric, the better the recommendation.

### 6.3. Model Comparison (RQ1)

To validate the effectiveness of our approach in combining preferences and health requirements, we compared the performance of FKGM with several baseline models in Top-N food recommendation, where ranking position K is set to 20. The experimental results are shown in Table 7.

**Figure 4:**
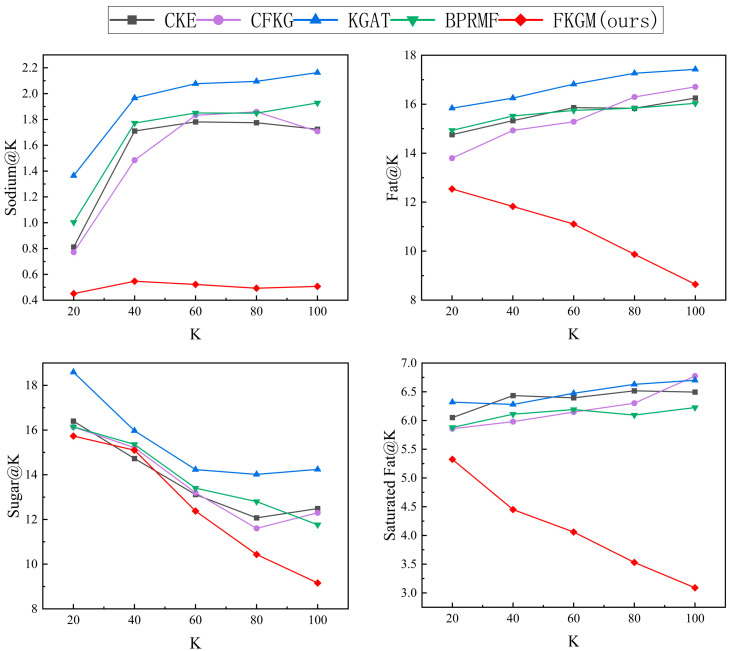
Comparison of performance of five models in health recommendation.

Table 7 shows the performance of FKGM and four baseline models in terms of the preference evaluation metric *Recall*@20 and four health evaluation metrics. The analysis shows that although FKGM did not achieve an optimal performance in dietary preference recommendation, it outperformed other models that only focus on preferences in terms of meeting user health requirements. A further analysis of the comparison between FKGM and other models in terms of health recommendation with the ranking parameter K set to {20, 40, 60, 80, 100} is shown in Figure 4.

After analyzing the experimental results shown in Figure 4, it can be concluded that our proposed model consistently outperformed other baseline models in health requirement recommendation, demonstrating the effectiveness of our approach in combining preference needs and health requirements.

### 6.4. Health Loss Weight Study (RQ2)

In this section, we study the impact of health loss weight on balancing preference needs and health requirements in food recommendations. By setting different health loss weights for experiments and comparing the model performance in health requirement recommendations, the results are shown in Table 8.

Experimental results show that with the increase of the health loss parameter $\lambda_{h}$, the performance of the model in health recommendation also increases. However, if $\lambda_{h}$ is too large, the performance of the model in dietary preference recommendation will be compromised. To balance the preference and health requirements of users in food recommendation, we suggest setting the health loss weight $\lambda_{h}$ to 0.1 for the model. Through experiments, it can be found that the model can control whether the recommendation results are more biased towards preferences or health requirements by adjusting the value of the health loss weight.

### 6.5. Hyper-Parameter Studies (RQ3)

To investigate the impact of different hyper-parameters on the performance of the FKGM model, we conducted multiple experiments and analyzed the performance of FKGM under different settings of embedding dimensions and message propagation layers.

#### 6.5.1. The Impact of FKGM Embedding Dimension

To investigate the impact of embedding dimensions on the performance of the FKGM model, we conducted multiple experiments. We set the embedding dimensions of the model to 8, 16, 32, 64, and 128, kept other parameters constant, and compared the experimental results. Table 9 shows the performance of FKGM under different embedding dimensions.

The experimental results indicate that with the increase in embedding dimensions, the model’s performance in preference recommendation first increases and then decreases. When the embedding dimension is set to 64, the model achieves the best performance in preference recommendation. In terms of healthy recommendation, the embedding dimension of 16 achieves the best performance in regulating sugar intake, while the other three nutrient constraints perform best when the embedding dimension is set to 64. Overall, the model performs best when the embedding dimension is set to 64.

#### 6.5.2. The Impact of the Number of Message Passing Layers

To investigate the impact of message passing layer depth on the performance of the FKGM model, we conducted experiments by setting the message passing layer depth to 1–5 and keeping other parameters constant. Table 10 presents the performance of FKGM under different layer depths.

The experimental results show that the performance of the FKGM model in preference recommendation initially increases with the depth of the message passing layer, but then starts to decrease. Stacking too many message passing layers does not always lead to an improvement in the recommendation performance of the model. This implies that overly deep message passing layers can lead to overfitting and loss of the model performance. Overall, the FKGM model achieved the best performance when the message passing layer depth was three.

## 7. Discussion

In this section, we discuss the main findings and implications of our proposed model FKGM. We also acknowledge the limitations of our study and suggest some directions for future research.

### 7.1. Main Findings and Implications

Our study aimed to address the issue of personalized healthy food recommendations by considering both user dietary preferences and health requirements. We constructed a collaborative recipe knowledge graph (CRKG) that contains user–recipe interactions and various food-related information. We also proposed a method for calculating the healthiness match between recipes and user preferences based on the Nutrient Content Score (NCS) of recipes and the Nutrient Intake Score (NIS) of users. To achieve this, we developed a novel health-aware food recommendation model (FKGM) that uses knowledge graph embedding and a knowledge-aware attention graph convolutional neural network to capture the semantic associations between users and recipes on CRKG, and learns the user’s requirements in both preference and health by fusing two losses of these two learning tasks.

We conducted experiments on the Allrecipes dataset and compared our model with four baseline models: CKE [22], CFKG [50], KGAT [23], and BPRMF [43], aiming to show that the FKGM model can learn users’ dietary preferences while considering their health requirements. The experimental results show that the FKGM model did not achieve the best performance in terms of preference; however, in the health recommendation task, our model achieved a significant advantage and outperformed the other baseline models. This suggests that our proposed FKGM can make balanced food recommendations in terms of preference and health requirements, rather than simply focusing on users’ preferences or the healthiness of the food itself. We believe that this advantage in terms of health has important practical application value in the future food recommendation field.

Furthermore, we conducted a study on the health loss weight in the multi-task learning layer, which directly affects the model’s performance on the preference recommendation and health recommendation tasks. The health loss weight can be adjusted to decide whether the model is more inclined to consider health or preference.

Our study has several implications for both research and practice. For research, our study contributes to the literature on food recommendation by proposing a new solution that integrates user preferences and health requirements. Our study also demonstrates the usefulness of knowledge graphs and multi-task learning techniques for food recommendation. For practice, our study provides a practical tool for food-related applications and services that aim to promote healthy eating habits among users. Our model can help users discover new recipes that suit their tastes and health requirements.

### 7.2. Limitations and Future Work

Despite the significant contributions of our study, we recognize certain limitations that require attention in future research. Firstly, we only considered four nutrients (sodium, fat, sugar, and saturated fat) as indicators of healthiness, which may not encompass all aspects of healthiness. Future research could integrate additional nutrients or other factors, such as calories, allergies, and dietary restrictions, in health assessments. Secondly, we evaluated our model using only one dataset (Allrecipes), which may restrict the generalizability of our results. Future research could apply our model to alternative domains or datasets, such as restaurant reviews or online grocery shopping. Thirdly, we did not conduct user studies or surveys to validate user satisfaction and acceptance of our model. Future research could gather user feedback or ratings to assess the user experience and perceived usefulness of our model.

## 8. Conclusions

This work applies food-related knowledge to food recommendation and proposes a healthy food recommendation model FKGM that considers both health requirements and user dietary preferences. FKGM is a knowledge graph-based multi-task learning model that learns semantic information between users and recipes through knowledge graph embedding and message-passing mefchanisms. It also emphasizes users’ unhealthy eating habits by learning their historical dietary behavior. This work constructed a large-scale collaborative recipe knowledge graph that contains user–recipe, recipe–ingredient, and other information for multi-task food recommendation, and extensive experiments were conducted on it. The results show that FKGM outperformed current competing baseline methods in the task of healthy food recommendation. The proposed health-aware food recommendation model is expected to have significant practical application value in the future food recommendation field.

## Figures and Tables

**Table 1:** Health threshold values for each nutrient in FSA rating.

Nutrient	Sodium	Fat	Sugar	Saturated Fat
**Threshold value (g/portion)**	0.7	21.0	27.0	6.0

**Table 2:** Examples of nutrient content in recipes (partial).

Recipe	Sodium (g)	Fat (g)	Sugar (g)	Saturated Fat (g)
Homemade Bacon	2.01	23.58	0.09	7.73
Foolproof Rosemary Chicken Wings	0.76	23.62	0.23	5.68
Cranberry Pork Chops II	0.40	6.31	26.46	2.73
Chinese Pot Roast	2.81	19.97	12.16	7.11

**Table 3:** Nutrient Content Scores calculated for the recipes in Table 2.

Recipe	*NCS_sodium_*	*NCS_fat_*	*NCS_sugar_*	*NCS_saturated fat_*
Homemade Bacon	28.81	11.23	0.03	12.90
Foolproof Rosemary Chicken Wings	10.90	11.25	0.09	9.47
Cranberry Pork Chops II	5.84	3.00	9.80	3.89
Chinese Pot Roast	40.14	9.51	4.50	11.86

**Table 4:** The NISs and the NCSs in case study.

k	Sodium	Fat	Sugar	Saturated Fat
${{NIS}_{k}(u}_{1})$	5.11	12.33	3.03	6.40
${{NIS}_{k}(u}_{2})$	10.39	4.50	13.14	6.24
${{NCS}_{k}(rp}_{1})$	10.90	11.25	0.09	9.47
${{NCS}_{k}(rp}_{2})$	4.63	5.80	11.38	5.51

**Table 5:** NDSs in case study.

${NDS(u}_{1},{rp}_{1})$	${NDS(u}_{1},{rp}_{2})$	${NDS(u_{2},rp}_{1})$	${NDS(u_{2},rp}_{2})$
74.55	16.40	44.51	68.99

**Table 6:** Hyper-parameters setting.

Parameter Name	Values	Parameter Name	Values
Embedding Dimension	64	Parameter_update	Adam
Message Passing Layer	3	Learning_rate	0.0001
Batch_size	2048	Message_dropout	0.1
Epoch	300	$\lambda_{h}$	0.1

**Table 7:** Performance of compared models.

Models	Test Set	High-Sodium	High-Fat	High-Sugar	High-Saturated Fat
	Recall@20	Sodium@20	Fat@20	Sugar@20	Saturated Fat@20
CKE	0.087	0.812	14.756	16.400	6.051
CFKG	0.086	0.772	13.799	16.414	5.858
KGAT	**0.090**	1.264	15.837	18.592	6.319
BPRMF	0.080	1.004	14.933	16.143	5.879
FKGM (ours)	0.074	**0.451**	**12.539**	**15.731**	**5.325**

**Table 8:** Performance comparison when FKGM sets different health loss weights.

Health Loss Weight	Recall@20	Sodium@20	Fat@20	Sugar@20	Saturated Fat@20
$\lambda_{h}=0$	**0.089**	1.167	16.186	17.567	6.316
$\lambda_{h}=0.05$	0.077	0.884	14.147	17.517	5.889
$\lambda_{h}=0.1$	0.074	0.451	12.539	15.731	5.325
$\lambda_{h}=0.15$	0.042	0.653	9.262	9.632	3.881
$\lambda_{h}=0.2$	0.032	**0.175**	**4.329**	**7.734**	**1.757**

**Table 9:** Performance comparison when model sets different embedding dimensions.

Dimension	Recall@20	Sodium@20	Fat@20	Sugar@20	Saturated Fat@20
**8**	0.055	1.026	15.793	17.011	6.181
**16**	0.063	0.735	15.855	**14.631**	6.293
**32**	0.071	0.812	14.756	18.382	5.850
**64**	**0.074**	**0.451**	**12.539**	15.731	**5.325**
**128**	0.067	0.571	13.331	16.235	5.855

**Table 10:** Performance comparison when model sets different message passing layers.

Layers	Recall@20	Sodium@20	Fat@20	Sugar@20	Saturated Fat@20
**FKGM-1**	0.063	0.698	14.815	17.950	5.688
**FKGM-2**	0.071	0.816	14.755	16.400	**5.188**
**FKGM-3**	**0.074**	**0.451**	**12.539**	**15.731**	5.325
**FKGM-4**	0.065	0.871	14.426	16.358	5.566
**FKGM-5**	0.056	1.060	15.299	17.414	6.117

## Data Availability

Data sharing is inapplicable for this article.
